# Trauma, Mental Health Workforce Shortages, and Health Equity: A Crisis in Public Health

**DOI:** 10.3390/ijerph22040620

**Published:** 2025-04-16

**Authors:** Suha Ballout

**Affiliations:** Manning College of Nursing and Health Sciences, University of Massachusetts Boston, Boston, MA 02125, USA; suha.ballout@umb.edu

**Keywords:** mental health workforce, burnout, secondary trauma, workforce resilience, trauma-informed care, equity, health disparities, workforce shortages

## Abstract

The global mental health workforce is facing a severe crisis marked by burnout, secondary trauma, compassion fatigue, and workforce shortages, with disproportionate effects on marginalized communities. This paper introduces the Integrated Workforce Trauma and Resilience (IWTR) Model, a comprehensive framework to understand and address these interconnected challenges. This study employs a conceptual, documentary analysis approach to examine the challenges faced by mental health workers, particularly trauma, burnout, and workforce shortages. By synthesizing existing qualitative and quantitative studies, the research identifies recurring themes and provides recommendations for policy reform to improve workforce sustainability and equity. Using a thematic synthesis of 75 peer-reviewed articles, conceptual papers, and policy reports published between 2020 and 2025, alongside foundational theoretical works, the IWTR Model integrates five theoretical perspectives: trauma-informed care, Conservation of Resources Theory, Intersectionality Theory, the Job Demands–Resources Model, and Organizational Justice Theory. The analysis identifies three dimensions: the impact of trauma on mental health professionals, organizational and systemic factors influencing workforce retention, and strategies to build resilience through policy and education. The findings reveal how secondary trauma, burnout, and systemic inequities interact to undermine workforce stability and access to care. The IWTR Model emphasizes that individual-level interventions will be insufficient without addressing structural issues, such as workload inequities, lack of leadership diversity, and underfunding. This model offers a roadmap for systemic reforms to strengthen workforce resilience, improve retention, and advance global equity in mental health care systems.

## 1. Introduction

The global mental health workforce is experiencing a severe crisis marked by widespread burnout, secondary trauma, and compassion fatigue, alongside critical workforce shortages and entrenched health inequities [1,2,3]. Between 21% and 67% of mental health professionals, including social workers, therapists, and psychiatric nurses, report burnout, often driven by high emotional demands and systemic stressors [4,5]. Compassion fatigue, similarly pervasive, affects 16% to 85% of clinicians working with trauma survivors, particularly those in acute care settings [6,7]. These workforce pressures are intensified by a global shortage of over 5 million mental health providers, disproportionately impacting low-income countries, rural areas, and marginalized communities [8,9,10]. The global mental health workforce is under immense pressure, with workforce shortages exacerbated by the increasing trauma experienced by both patients and health care providers. Health care workers, especially those in high-need environments, are exposed to the trauma of their patients, which leads to secondary traumatic stress (STS) and burnout. These effects, in turn, contribute to workforce attrition, creating a cyclical crisis [11,12]

Mental health workforce shortages are evident worldwide. In the United States, over 169 million people live in federally designated Mental Health Professional Shortage Areas (MHPAs), with Black, Indigenous, and people of color (BIPOC) communities facing the most significant gaps in care [13,14]. The lack of workforce diversity in mental health services also exacerbates these challenges. Studies show that 86% of psychologists and 88% of mental health counselors in the US are white, limiting the cultural competence of care and hindering access for underrepresented populations [15,16]. These shortages and structural inequities demand urgent systemic reforms to address workforce retention, equity, and diversity in mental health care. Canada faces similar challenges, with patients in some regions waiting over six months for psychiatric care [9]. In the United Kingdom, more than 70% of mental health professionals report severe emotional exhaustion, contributing to high turnover rates [7,8]. In Australia, rural and Indigenous populations face critical shortages, relying heavily on emergency services and telehealth [17]. In sub-Saharan Africa, there are fewer than 1 psychiatrist per 500,000 people, while in India the psychiatrist to population ratio is just 0.75 per 100,000 [10,15,18]. Latin American nations struggle with low wages and high job stress, pushing many mental health workers out of public service or into migration [16]. These global examples highlight the need for urgent policy action and sustainable workforce strategies [19]. Deepening these shortages, mental health providers, psychiatrists, psychologists, social workers, and psychiatric nurses face moral injury when systemic barriers, such as unmanageable caseloads and inadequate institutional support, prevent them from delivering quality care [9,11,20,21]. The COVID-19 pandemic further heightened these pressures, driving widespread distress, burnout, and workforce attrition [12]. These challenges significantly restrict access to care and deepen global inequities in mental health services [1,7].

Despite substantial research, many existing frameworks approach burnout, secondary trauma, and workforce shortages as isolated issues, failing to address their interconnected nature [22,23]. To address this gap, this paper introduces the Integrated Workforce Trauma and Resilience (IWTR) Model, an innovative framework that draws on trauma-informed care, Conservation of Resources Theory, Intersectionality Theory, the Job Demands–Resources Model, and Organizational Justice Theory. By focusing on systemic, policy-driven solutions rather than individualized approaches alone, the IWTR Model offers a novel lens for addressing workforce trauma and sustainability [12,23,24,25,26]. This paper aims to guide policy, workforce education, and organizational reform to strengthen mental health workforce retention, resilience, and equity [27,28]. This research aims to explore the challenges faced by mental health workers and the systemic reforms needed to address workforce trauma, burnout, and inequities. As a conceptual study using documentary analysis, the research synthesizes the existing literature, including qualitative and quantitative studies, to identify key themes and provide actionable policy and practice reform recommendations.

The global mental health workforce is facing acute challenges, with disparities in workforce distribution and access to care being particularly pronounced in rural and underserved regions. For example, Australia’s rural mental health professionals struggle with high caseloads and emotional exhaustion, necessitating telehealth and trauma-informed care approaches [17]. In India, the shortage of mental health professionals is compounded by a low psychiatrist to population ratio, highlighting the need for innovative workforce solutions [15]. Similarly, Latin American countries face workforce migration trends, further deepening the crisis in public mental health services [16]. These examples demonstrate that workforce shortages and structural inequities are global issues requiring tailored solutions and comprehensive frameworks like the IWTR Model.

## 2. Background

### 2.1. Multidimensional Trauma in the Mental Health Workforce

Mental health professionals are increasingly vulnerable to multidimensional trauma, including secondary traumatic stress (STS), burnout, compassion fatigue, and moral distress, which are deeply intertwined with systemic failures in mental health care delivery [2,7]. Unlike primary trauma, STS arises from continuous exposure to clients’ traumatic experiences, leading to symptoms that mirror post-traumatic stress disorder (PTSD), such as hypervigilance, emotional numbing, and intrusive thoughts [29]. STS prevalence rates vary widely, with up to 39% of mental health professionals, including social workers, psychiatric nurses, and juvenile justice workers, experiencing significant symptoms depending on their settings [11]. The COVID-19 pandemic amplified these risks as providers faced heightened exposure to patient suffering, increased mortality rates, and unrelenting emotional labor in under-resourced environments [12].

Burnout, a distinct but often overlapping phenomenon with STS, is marked by chronic emotional exhaustion, depersonalization, and a diminished sense of professional efficacy [3,22]. Mental health workers, particularly those in high-demand fields, such as community mental health and hospital settings, face excessive caseloads, administrative burdens, and systemic stressors that significantly contribute to emotional exhaustion and professional disengagement [1,5]. The Job Demands–Resources (JD-R) Model showcases how persistent high job demands coupled with insufficient organizational support accelerate burnout and lead to increased workforce attrition [22,30]. Studies suggest that burnout is widespread, with early-career mental health professionals at exceptionally high risk due to inadequate supervision, training, and peer support structures [31]. Also, structural inequities exacerbate these conditions. Racially and ethnically minoritized mental health professionals, including Black, Indigenous, and LGBTQ+ clinicians, report higher levels of racial traumatic stress, workplace discrimination, and cultural taxation, which compound their risk of burnout and moral distress [14]. These providers often bear the dual burden of managing their clients’ trauma while also navigating institutional racism, microaggressions, and exclusion from leadership pathways, factors that contribute to emotional depletion and workforce attrition [16,19].

Compassion fatigue, another critical form of workforce trauma, refers to the emotional and physical exhaustion resulting from prolonged exposure to clients’ suffering. Compassion fatigue affects between 16% and 85% of mental health professionals, especially emergency department nurses, crisis workers, and community-based clinicians serving highly vulnerable populations [6,7]. The absence of robust organizational support systems exacerbates these issues, leading to higher absenteeism rates, reduced job satisfaction, and professional withdrawal [9,20]. Also, research indicates that clinicians with personal trauma histories are more prone to developing vicarious trauma and moral injury, further increasing their likelihood of exiting the workforce [21,32,33]. Mental health providers are often expected to deliver trauma-informed care to clients without receiving similar support or trauma-responsive practices within their organizations [34]. Given these alarming trends, system-level interventions are urgently needed to prevent workforce depletion and ensure the sustainability of high-quality, trauma-informed mental health services [24,28]. Addressing these issues requires integrated policies emphasizing organizational justice, reducing occupational stressors, and embedding workforce resilience strategies into institutional frameworks [12,23].

### 2.2. Growing Workforce Shortages and Disparities in Access

Global workforce shortages significantly limit access to mental health care, with demand far exceeding supply in nearly every region [1,9]. The World Health Organization [10] reports that nearly 50% of the global population lives in countries with fewer than 1 psychiatrist per 100,000 people, highlighting critical inequities in mental health access [12]. These shortages are particularly severe in low-income countries, where primary mental health services are often unavailable [8]. For instance, in sub-Saharan Africa, there is less than 1 psychiatrist per 500,000 people, and in India only 0.75 psychiatrists are available per 100,000 individuals [10,18]. As a result, general health care workers with limited mental health training are tasked with meeting the complex needs of individuals with serious mental illness [15]. In high-income countries, access remains severely unequal. Over 169 million people in the United States live in federally designated Mental Health Professional Shortage Areas (MHPAs), with disproportionately high gaps in care for Black, Indigenous, and people of color (BIPOC) communities [13,14]. Canada faces similar shortages, with psychiatric wait times exceeding six months in many regions [9]. In Australia, rural and Indigenous populations rely heavily on emergency departments and telehealth due to a lack of psychiatric specialists [17]. Meanwhile, in the United Kingdom, over 70% of mental health workers report severe emotional exhaustion, a factor that contributes to rising attrition [7,8]. Latin American countries experience workforce depletion due to low wages and poor working conditions, which push skilled professionals into private practice or migration for better opportunities [16].

The COVID-19 pandemic further intensified workforce shortages as demand for mental health services spiked alongside rising rates of burnout and early retirement among clinicians [2,3]. Surveys consistently identify low pay, unsustainable caseloads, and lack of institutional support as leading causes of workforce attrition [13,21,35,36,37]. These shortages disproportionately affect marginalized and rural communities, worsening disparities in mental health care access [1,9]. Also, diversity in the mental health workforce remains starkly lacking. Nearly 86% of psychologists and 88% of mental health counselors in the US are white, creating significant barriers to culturally responsive care for BIPOC clients [14]. Without diverse providers, patients from underrepresented groups are less likely to seek care, perpetuating disparities in access and outcomes [6,23]. Also, many training programs still neglect to incorporate robust diversity education, leaving clinicians underprepared to deliver culturally competent services [7,24]. Thus, workforce shortages, combined with a lack of diversity and structural inequities, undermine the ability of mental health systems to meet rising demands. Addressing these challenges requires urgent policy reforms, expanded workforce development initiatives, and targeted investments in retention and training [8,9]. The Integrated Workforce Trauma and Resilience (IWTR) Model offers a framework for understanding and addressing these interconnected issues, emphasizing systemic solutions to mitigate burnout, improve retention, and advance global mental health workforce equity.

## 3. Methods

This study employs a conceptual, documentary analysis approach using a thematic synthesis method to analyze the existing literature. Unlike traditional qualitative research involving direct participant interaction, this research synthesizes qualitative and quantitative studies to identify recurring themes and patterns from various sources. This method allows for a comprehensive understanding of the issues surrounding mental health workforce trauma, burnout, and equity drawn from diverse, pre-existing studies. A thematic synthesis approach was employed to guide the development of the IWTR Model by reviewing 75 peer-reviewed articles, conceptual papers, and policy reports published between 2020 and 2025 alongside foundational theoretical work. We conducted a literature search using PubMed, PsycINFO, Scopus, and Web of Science. Inclusion criteria included peer-reviewed articles published between 2020 and 2025 that addressed mental health workforce challenges, such as burnout, secondary trauma, systemic inequities, and resilience. Studies not published in English or unrelated to the mental health workforce were excluded. The selected literature was coded iteratively and organized using a thematic synthesis approach based on theoretical relevance. These sources were coded to identify recurring patterns and mechanisms related to workforce trauma, burnout, secondary traumatic stress, structural inequities, and resilience strategies. The identified themes were organized around five guiding theoretical frameworks, trauma-informed care (TIC) [38], Conservation of Resources (COR) Theory [39], Intersectionality Theory [40], the Job Demands–Resources (JD-R) Model [30], and Organizational Justice Theory [41], allowing for a multidimensional understanding of workforce challenges and solutions. To ensure analytic rigor and conceptual validity, the emerging themes and dimensions of the IWTR Model were reviewed and refined iteratively through internal team discussions, triangulation with seminal studies, and cross-referencing with key policy reports. Although a formal peer review of the thematic synthesis was not conducted, the analysis was closely aligned with established findings in workforce trauma, health equity, and trauma-informed care research, ensuring conceptual clarity, relevance, and scholarly rigor. This process ensured that the IWTR Model reflects theoretical insights, current empirical evidence, and policy priorities, offering a comprehensive, evidence-informed framework for guiding workforce resilience, retention, and equity interventions in mental health care.

### 3.1. Development of the IWTR Model

**Figure 1:**
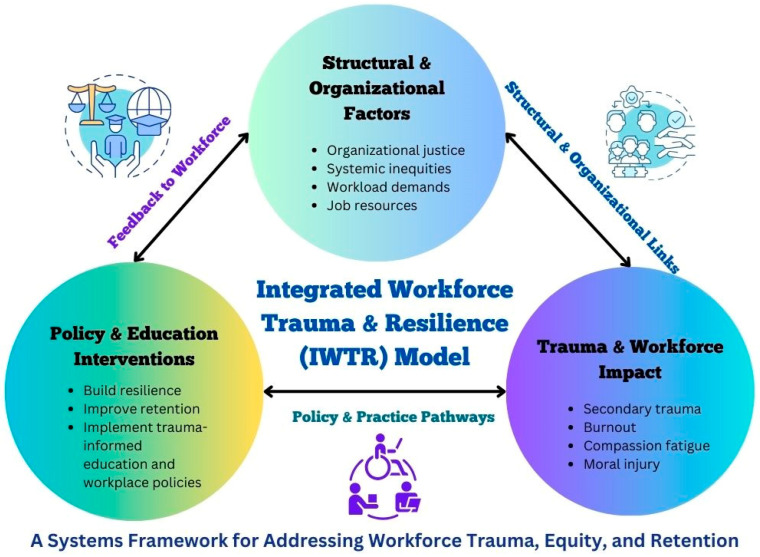
This figure visually represents the IWTR Model, with its three dimensions depicted as interlocking components. A unique color band maps corresponding stressors and interventions for each framework. Trauma-informed care and Conservation of Resources inform the trauma and resilience pathways; the JD-R Model and Organizational Justice highlight structural contributors to burnout; Intersectionality Theory frames how inequities intensify these effects across provider groups.

The IWTR Model was developed through an integration of five well-established theoretical perspectives: trauma-informed care (TIC), Conservation of Resources (COR) Theory, Intersectionality Theory, the Job Demands-Resources (JD-R) Model, and Organizational Justice Theory [30,38,39,40]. These frameworks were analyzed to identify key themes and mechanisms influencing workforce trauma, burnout, and retention [2,3,31]. A thematic synthesis approach was used to categorize recurring patterns in the literature by mapping the interconnections between workplace demands, systemic inequities, organizational support, and workforce outcomes [1,5,9]. This integrative process enabled the creation of a multidimensional model to guide workforce resilience strategies [24,42]. Because this project is conceptual and does not involve human subjects, institutional ethical approval was not required. Nonetheless, all sources are fully cited, ensuring academic rigor and intellectual integrity [22,31]. The final IWTR Model (Figure 1) aims to offer an evidence-informed framework that policymakers, educators, and health care leaders can apply to design systemic interventions that improve workforce resilience, retention, and equity in mental health care [12,26,28].

### 3.2. The Integrated Workforce Trauma and Resilience (IWTR) Model

The IWTR Model addresses the interconnected challenges of trauma, burnout, workforce shortages, and systemic inequities that affect mental health professionals. Drawing on interdisciplinary theories, the IWTR Model offers an equity-centered framework for understanding workforce trauma and designing systemic solutions [1,2,12,31].

The trauma-informed care (TIC) framework emphasizes that mental health professionals, like their patients, require trauma-responsive workplace environments to prevent secondary traumatic stress and compassion fatigue [2,7,11,25,43]. However, many mental health organizations lack structures that acknowledge providers’ exposure to trauma, leading to deep emotional distress [6,7,13]. TIC calls for psychological safety, trust, peer support, and institutional self-care, advocating for structural interventions rather than over-relying on individual coping strategies [31].

Conservation of Resources (COR) Theory complements TIC by explaining burnout due to chronic resource depletion, including the erosion of emotional, cognitive, and professional capacities [22,32,43]. COR Theory highlights how ongoing resource loss, stemming from underfunding, insufficient staffing, and overwhelming caseloads, fuels emotional exhaustion and workforce attrition [6,9]. Interventions to replenish resources, such as mentorship, leadership development, and reduced workloads, are central to sustaining workforce capacity [36,37].

Recognizing that workforce trauma is unequally distributed, Intersectionality Theory provides a lens to understand how systemic discrimination, racism, and cultural taxation shape the experiences of marginalized providers [2,14,16,40]. Black, Indigenous, and LGBTQ+ clinicians face racial microaggressions, workplace exclusion, and higher emotional labor demands, exacerbating burnout and increasing turnover [26]. The model highlights the need for equity-driven workforce policies that center on diversity, equity, and inclusion (DEI) to address these layered stressors [7,21].

The Job Demands–Resources (JD-R) Model demonstrates how excessive job demands (e.g., high caseloads, emotional labor, lack of autonomy) and insufficient organizational resources contribute to burnout and attrition [3,22]. Increasing job resources, such as clinical supervision, flexible scheduling, and professional growth opportunities, improves retention and reduces burnout [9,23,26,43]. Research shows that organizations that invest in workforce support experience lower attrition and higher morale [5,8].

Finally, Organizational Justice Theory emphasizes that perceptions of fairness, leadership transparency, and equitable policies influence workforce morale and retention [36,41,44]. Unfair workload distributions, opaque leadership decisions, and lack of advancement opportunities foster disengagement, emotional exhaustion, and exit intentions [20,22]. Conversely, organizations that promote shared governance, pay equity, and inclusive leadership foster resilience and retention [12,24,28]. As shown in Table 1, the integration of trauma-informed care and Conservation of Resources Theory in the first dimension highlights the importance of both individual and systemic factors in addressing trauma exposure among mental health professionals.

Through iterative analysis of interdisciplinary theories and the workforce literature, the IWTR Model emerges as a holistic, multidimensional approach to workforce resilience [22,31]. By integrating trauma-informed, organizational, and equity-focused perspectives, the IWTR Model shifts the focus from individual coping to system-level reforms that address burnout, support retention, and advance health equity in mental health care [1,18,27,32,44].

## 4. Results

The Integrated Workforce Trauma and Resilience (IWTR) Model offers a multidimensional understanding of the forces driving workforce trauma, burnout, and retention challenges in mental health care. Synthesizing five theoretical frameworks, trauma-informed care (TIC), Conservation of Resources (COR) Theory, Intersectionality Theory, the Job Demands–Resources (JD-R) Model, and Organizational Justice Theory, the IWTR Model identifies three interrelated dimensions: (1) The Impact of Trauma on the Mental Health Workforce, (2) Structural and Organizational Factors Affecting Workforce Retention, and (3) Building Workforce Resilience Through Policy and Education. To provide further clarity regarding these dimensions, Table 1, Table 2 and Table 3 summarize the key constructs from the articles reviewed for each dimension, offering a comparative view of the variables and recommendations drawn from the literature. These dimensions show how systemic inequities, organizational stressors, and chronic under-resourcing converge to create widespread workforce distress and offer a roadmap for systemic interventions.

### 4.1. The Impact of Trauma on the Mental Health Workforce (IWTR Dimension 1)

The IWTR Model identifies that trauma within the mental health workforce is not merely a product of individual patient encounters but compounded by structural inequities and chronic organizational failures. Mental health professionals, especially those in community-based, psychiatric, and crisis response roles, face persistent exposure to secondary traumatic stress (STS), moral injury, and compassion fatigue [7,11]. The model emphasizes how unrelenting exposure to patient trauma, coupled with a lack of institutional supports, such as peer debriefing and supervision, lead to emotional exhaustion and disengagement, with many providers reporting PTSD-like symptoms [29,45]. The first dimension of the IWTR Model, which examines the impact of trauma on mental health professionals, is demonstrated in countries like Australia, where rural mental health workers face high levels of burnout and secondary trauma due to a lack of professional support and high caseloads [17]. Similarly, in India, mental health professionals are under severe stress due to a low psychiatrist to population ratio, which exacerbates burnout and limits their ability to provide comprehensive care [15]. These global examples underscore the need for targeted policy reforms and support systems tailored to the specific needs of each workforce.

Psychiatric nurses and community mental health workers are particularly vulnerable given their dual role in providing clinical care while navigating environments with high violence risks, ethical dilemmas, and insufficient staffing [6,24]. The COVID-19 pandemic significantly deepened these conditions, increasing patient acuity and mortality while eroding provider support systems [1,12]. COR Theory emphasizes how this cumulative emotional labor leads to resource depletion, accelerating burnout and driving workforce attrition [22,26,43]. The IWTR Model draws on Intersectionality Theory to show how structural inequities disproportionately impact marginalized providers. Black, Indigenous, and LGBTQ+ clinicians face layered stressors, including racial discrimination, microaggressions, and cultural taxation (being expected to serve as cultural brokers without additional support) [14,26]. These providers often work in underfunded, high-need environments and are excluded from leadership opportunities, contributing to higher burnout and turnover rates [16,27]. Structural racism exacerbates these disparities, as evidenced by pay inequities and a lack of promotion pathways [15,18].

Marginalized demographic groups disproportionately feel the impact of workforce shortages. In the US, over 169 million people reside in Mental Health Professional Shortage Areas (MHPAs), with the highest concentration in BIPOC communities [13,14]. Additionally, racial disparities persist in the mental health workforce itself, with 86% of psychologists and 88% of mental health counselors identifying as white [16]. These figures underscore the need for targeted policies addressing workforce shortages while prioritizing diversity and inclusivity in leadership opportunities. The impact of workforce shortages on access to mental health care is particularly severe in rural and underserved areas. To mitigate these challenges, telehealth has emerged as an alternative solution to expand access to care. Remote consultations via telehealth platforms have shown potential in addressing the gap in mental health services. However, challenges remain, such as digital inequity, with marginalized populations often lacking access to necessary technology and the need for trauma-informed virtual care to ensure the quality of services.

As highlighted by the IWTR Model, the combination of vicarious trauma, organizational neglect, and systemic discrimination creates a high-risk environment for emotional exhaustion and workforce depletion, requiring trauma-responsive and equity-centered interventions to prevent ongoing attrition [28,40]. The IWTR Model highlights the critical relationship between trauma exposure and workforce attrition. Health care workers, especially those in trauma-intensive settings, face significant emotional tolls as a result of repeated exposure to patients’ trauma. Secondary traumatic stress (STS), burnout, and compassion fatigue are prevalent, particularly in environments where workers lack adequate support [11,12]. Conservation of Resources Theory offers a lens through which to understand this dynamic, as it illustrates how persistent trauma exposure drains essential emotional, cognitive, and professional resources. These resources are depleted without sufficient organizational support and coping mechanisms, leading to burnout and workforce turnover [6].

### 4.2. Structural and Organizational Factors Affecting Workforce Retention (IWTR Dimension 2)

The IWTR Model emphasizes that organizational and structural conditions are pivotal in shaping workforce retention. Drawing on the JD-R Model and Organizational Justice Theory, this dimension focuses on how job demands, resource scarcity, and perceived unfairness contribute to workforce instability.

Mental health professionals, particularly those in public sector and community-based roles, face excessive caseloads, long hours, and administrative overload, with insufficient institutional resources to balance these demands [2,11]. The JD-R Model shows that burnout and disengagement escalate when high job demands are not met with adequate resources, such as clinical supervision, fair wages, and mental health support [5,22]. Administrative burdens, including complex documentation and billing requirements, are frequently cited as significant contributors to professional dissatisfaction and intent to leave the field [21,27]. Also, Organizational Justice Theory shows that perceptions of unfairness in pay, promotion opportunities, and workload distribution directly impact workforce morale and retention [15,41,44]. Mental health professionals working in community and public health sectors earn significantly less than hospital-based and private practice peers despite managing higher-acuity populations [16,18]. This wage disparity is a major driver of workforce turnover, particularly in high-demand areas [1,9]. Also, racial and gender inequities in career advancement and leadership opportunities remain deeply entrenched. Black, Indigenous, LGBTQ+, and immigrant clinicians often encounter systemic barriers to leadership, further compounding job dissatisfaction and fueling workforce attrition [14,35]. The IWTR Model highlights that failure to address these inequities undermines organizational cohesion, limits culturally responsive care, and perpetuates workforce shortages [26].

Without system-wide reforms, including equitable pay structures, reduced job demands, and pathways for leadership advancement, the cycle of burnout and attrition will continue to destabilize the mental health workforce [9,19].

### 4.3. Building Workforce Resilience Through Policy and Education (IWTR Dimension 3)

The IWTR Model identifies systemic education, workplace policies, and policy reforms as essential to fostering long-term workforce resilience. Trauma-informed care (TIC) and COR Theory emphasize that workforce well-being must be embedded in training and organizational culture to prevent emotional exhaustion and sustain professional capacity [20,46]. Integrating trauma-informed content into nursing, social work, and mental health training programs can equip future professionals to manage emotional labor and prevent burnout [7,11]. The IWTR Model supports secondary trauma and resilience training, peer support and debriefing programs, and mindfulness interventions as part of standard curricula [2,8,31]. These measures are essential to strengthening providers’ capacity to navigate their roles’ emotional challenges and reduce isolation, a key contributor to workforce distress [12,22].

Workplace policies grounded in TIC and Organizational Justice Theory are also essential. Trauma-responsive leadership training for health care managers is key to fostering supportive environments and addressing secondary trauma proactively [9,19]. Equitable pay, transparent workload distribution policies, and structured debriefing and peer support programs can mitigate burnout and promote organizational loyalty [26,36,46]. Finally, legislative reforms aligned with the JD-R Model and Organizational Justice Theory are necessary to address systemic barriers to workforce sustainability. Policy solutions include increasing salaries for community-based mental health professionals, expanding loan forgiveness and retention bonuses, and implementing safe staffing mandates to prevent overburdening [5,16,21]. Without such legislative action, organizations will continue to struggle with recruitment and retention, limiting access to care in underserved communities [1,15]. Also, creating leadership pathways for marginalized professionals, such as Black, Indigenous, LGBTQ+, and immigrant clinicians, remains critical to fostering inclusive, equitable workplaces and ensuring culturally responsive care [14,16,26].

The three dimensions of the IWTR Model, the impact of trauma on the mental health workforce, structural and organizational factors affecting workforce retention, and building workforce resilience through policy and education, illustrate how workforce trauma, burnout, and inequities are interconnected. Table 2 provides a comparative overview of the policy recommendations for workforce sustainability. It describes how loan forgiveness and safe staffing mandates are consistently supported across the studies to mitigate workforce shortages and improve retention rates. Trauma exposure and resource depletion contribute to burnout and attrition, while structural and organizational barriers exacerbate these issues by limiting support, fair compensation, and career advancement opportunities. Without systemic interventions, these challenges perpetuate workforce shortages and deepen disparities in access to care. The IWTR Model emphasizes that addressing workforce trauma requires a multi-level approach combining trauma-informed education, organizational justice, and policy reforms to create sustainable, equitable, and resilient mental health systems. This synthesis sets the foundation for actionable strategies discussed in the following section.

## 5. Discussion

The findings of this project provide a critical lens for understanding and addressing the intertwined crises of workforce trauma, shortages, and inequities in the mental health sector. The IWTR Model’s three dimensions, trauma’s impact on the workforce, structural and organizational factors influencing retention, and pathways to resilience through policy and education, offer a comprehensive, multidimensional understanding of mental health professionals’ challenges and the urgent need for systemic reforms.

First, the IWTR Model highlights that trauma experienced by mental health professionals is not merely individual but structurally embedded. Workforce trauma, manifested as secondary traumatic stress (STS), compassion fatigue, burnout, and moral injury, is a product of systemic failures to protect and support providers exposed to high-risk and emotionally demanding environments [7,11]. The model’s integration of trauma-informed care (TIC) and Conservation of Resources (COR) Theory highlights that emotional exhaustion arises when organizations fail to replenish workers’ emotional and professional resources [6,22,47]. Without access to peer support, debriefing opportunities, or trauma-responsive leadership, many providers experience unmitigated stress, leading to emotional disengagement and attrition [2,12]. These findings align with research showing that frontline mental health professionals, especially in community and crisis settings, experience higher rates of burnout and workforce exit when operating under resource-constrained environments [3,5]. Nurses and social workers who serve high-need populations report exceptionally high levels of burnout and STS when faced with insufficient organizational support [6,24]. Therefore, interventions prioritizing trauma-informed organizational cultures and reducing chronic resource depletion are critical to stabilizing the workforce [1,9].

The disparities highlighted by the empirical data reinforce the urgent need for a multi-level approach to addressing workforce trauma and shortages. As the data show, BIPOC communities face significantly greater gaps in mental health care access due to mental health professional shortages [13,14]. Additionally, the overrepresentation of white professionals in mental health roles and the lack of diversity in leadership exacerbate these challenges, leading to suboptimal care for underrepresented populations [16]. These findings highlight the importance of the IWTR Model’s emphasis on equity-driven interventions, which must include addressing workforce shortages, increasing diversity, ensuring equitable pay, and expanding leadership opportunities for marginalized providers.

A significant contribution of the IWTR Model is its incorporation of Intersectionality Theory, which foregrounds how workforce trauma and burnout disproportionately impact marginalized providers, including Black, Indigenous, LGBTQ+, and immigrant clinicians [14,40]. These professionals face not only the stress of high patient caseloads but also systemic barriers, such as racial discrimination, exclusion from leadership, and cultural taxation (being expected to offer culturally competent care without institutional recognition or support) [15,16,19,48]. These findings resonate with broader studies highlighting how systemic racism and inequity exacerbate workforce burnout, particularly for racially minoritized professionals [15,18]. Providers from marginalized communities are also overrepresented in underfunded, high-burden clinical settings, further increasing their vulnerability to burnout and workforce exit [7,26]. The IWTR Model’s intersectional analysis calls for urgent, equity-centered workforce strategies that address the emotional demands of clinical work and the structural injustices embedded in the health care system [21,34,36,49].

The connection between patient trauma and workforce shortages is rooted in the emotional and psychological toll experienced by health care providers. In trauma-intensive settings, workers are exposed to the traumatic experiences of their patients, leading to secondary traumatic stress (STS), burnout, and compassion fatigue. These effects are compounded when there is a lack of institutional support and trauma-informed resources for providers [11,12]. The Conservation of Resources Theory explains how continuous exposure to trauma without adequate replenishment of personal and professional resources can lead to emotional depletion, causing health care workers to exit the field and further exacerbating workforce shortages [6]. This principle highlights the critical need for trauma-informed care systems that not only support patient needs but also prioritize the resilience and well-being of health care workers, thereby improving retention and reducing workforce turnover.

The second dimension of the IWTR Model, focusing on structural and organizational factors, reinforces that workforce retention is profoundly shaped by organizational justice, fairness, and adequate resourcing. Consistent with Organizational Justice Theory, the model highlights that inequities in pay, promotion opportunities, and workload distribution erode morale and drive workforce attrition [21,41,50]. These findings are supported by studies documenting that community-based and public sector mental health workers who serve the most vulnerable populations are often paid less than their counterparts in private or hospital settings despite carrying heavier caseloads and facing greater emotional demands [9,16]. Also, persistent pay inequities and lack of career advancement disproportionately impact clinicians from marginalized backgrounds, reinforcing patterns of exclusion and burnout [14,15]. The IWTR Model emphasizes the need for organizations to implement policies that ensure equitable pay, transparent promotion pathways, and leadership opportunities for underrepresented providers [26,36]. Without addressing these organizational justice issues, mental health systems will lose diverse, experienced professionals essential to culturally competent care [8,28].

Telehealth has increasingly been recognized as a key strategy to address mental health workforce shortages, particularly in rural and underserved regions. By offering remote consultations, telehealth expands access to mental health services, mitigating the effects of workforce gaps. However, this solution is not without its challenges. Digital inequity, such as limited access to broadband internet and devices, disproportionately affects marginalized communities, limiting the accessibility of telehealth services. Furthermore, the implementation of trauma-informed care within virtual settings requires specific attention. Providers must receive trauma-informed training to adapt their practices to the virtual environment and address the unique needs of trauma survivors in this format. Addressing these barriers requires targeted investments in technology infrastructure, training, and policy development, particularly in regions where the digital divide is the workforce shortage that most impacts pronounced and marginalized populations. In this context, telehealth must be integrated into a broader, equity-driven framework for mental health care to ensure that both access and provider resilience are enhanced.

The third dimension of the IWTR Model emphasizes systemic reforms through trauma-informed education, organizational policy, and legislation as essential for building a sustainable workforce. The integration of TIC and COR Theory highlights that educational institutions play a critical role in preparing providers for the emotional realities of mental health work [31,46]. Embedding trauma-informed curricula, including training on secondary trauma, resilience strategies, and peer debriefing, is essential to reducing early-career burnout and improving retention [7,11]. Educational reforms prioritizing provider well-being and clinical competence ensure that future professionals are equipped for long-term careers [27]. In addition to education, workplace policies that align with TIC principles, such as structured debriefing programs, trauma-responsive leadership, and equitable workload distribution, are key to sustaining the workforce [9,19]. Also, legislative actions guided by the JD-R Model and Organizational Justice Theory are necessary to address systemic barriers, including underfunding, unsafe staffing ratios, and inequitable compensation [22,30,42]. Increasing salaries, expanding loan forgiveness, and enforcing safe staffing mandates are key strategies to mitigate burnout and improve workforce retention [5,16].

While the IWTR Model emphasizes the need for trauma-informed care, organizational justice, and workforce resilience, these recommendations are most effective when supported by existing and proposed policies. For instance, the US Department of Health and Human Services has proposed mental health workforce development initiatives to expand and diversify the workforce, which aligns with the model’s focus on leadership development and cultural competency training [51]. Policies like loan forgiveness programs for mental health professionals working in underserved areas can address workforce shortages and support retention in high-need regions [4]. Moreover, safe staffing mandates proposed by health care advocacy organizations are essential to ensure that the mental health workforce is not overburdened. This directly ties into the model’s emphasis on reducing burnout and improving care quality [1]. These policy measures, alongside global frameworks from the World Health Organization (WHO) on workforce resilience, provide a concrete foundation for implementing the recommendations outlined in the IWTR Model. Table 3 summarizes the implementation barriers and solutions identified in the literature. For example, while financial constraints remain a significant barrier, several studies propose targeted funding initiatives and policy reforms to address these challenges.

While the IWTR Model provides a conceptual framework for understanding workforce trauma, burnout, and inequities, comparative data and case studies can further support its practical application. For instance, in Australia, rural mental health challenges are exacerbated by workforce shortages and a heavy reliance on telehealth to reach remote communities, illustrating the need for infrastructure development and trauma-informed virtual care [17]. Similarly, in India, there is a critical shortage of mental health professionals, with fewer than 0.75 psychiatrists per 100,000 people, which highlights the urgent need for workforce expansion and culturally competent training [15]. Additionally, Latin America faces significant workforce migration trends, with many skilled professionals leaving the public sector for private practice or migrating for better opportunities, thus deepening workforce shortages [16]. These case studies illustrate the model’s global applicability and underscore the importance of context-specific solutions in addressing workforce resilience and equity in diverse settings. The IWTR Model provides a framework for policymakers, educators, and health care leaders to develop comprehensive, systemic interventions beyond individual coping strategies to address structural determinants of workforce trauma and burnout. For policymakers, this model stresses the need for sustained investment in mental health workforce development, including targeted incentives for underserved areas and marginalized professionals [9,15]. For educational institutions, it calls for integrating trauma-informed pedagogy that prepares clinicians for the emotional complexity of mental health care [11,31]. For organizations, it demands a shift toward trauma-responsive leadership, organizational justice, and the creation of supportive, inclusive workplaces [26,42].

## 6. Limitations and Future Research

While the IWTR Model offers a conceptual contribution grounded in a rigorous literature synthesis, this study does not include primary data or empirical testing. Potential limitations include publication bias, excluding non-English sources, and limited regional contextualization. Future research should empirically test the model in diverse settings, conduct longitudinal studies on interventions, and develop comparative case studies across countries and systems.

## 7. Conclusions

The IWTR Model offers an innovative approach to understanding and addressing the multifaceted challenges of workforce trauma, shortages, and inequities in mental health care. This research has successfully met its objective by developing a conceptual framework that integrates trauma, burnout, systemic inequities, and workforce policy through a thematic synthesis of scholarly and policy literature. The IWTR Model provides an understanding of the interconnected challenges in the mental health workforce and proposes actionable, systemic reforms to support workforce resilience. By integrating trauma-informed care, resource conservation, intersectionality, organizational justice, and the Job Demands–Resources framework, the model underscores the critical need for systemic, policy-driven interventions to foster sustainable workforce resilience. The findings from the analysis of the three dimensions—(1) the impact of trauma on the mental health workforce, (2) structural and organizational factors affecting workforce retention, and (3) building workforce resilience through policy and education—highlight the crucial role of public policy in addressing workforce shortages, burnout, and systemic inequities. Specifically, policies like safe staffing mandates, loan forgiveness programs, equitable pay structures, and targeted legislative support for marginalized providers are essential to ensuring long-term retention and advancing health equity within mental health systems. Also, this research emphasizes that the impact of trauma, compounded by inadequate organizational support, creates a cycle of burnout and workforce attrition. To combat this, health systems must prioritize trauma-responsive education frameworks, implement equitable workplace practices, and enact comprehensive legislative reforms. Specifically, policy actions should focus on expanding workforce funding, improving safe staffing mandates, and creating leadership development programs that are inclusive of marginalized groups. Additionally, investments in mental health workforce training, particularly in trauma-informed care and culturally responsive practices, are crucial to sustain the profession’s growth and ensure providers are prepared for the emotional demands of their roles.

The IWTR Model synthesizes theoretical insights and policy priorities and offers a clear roadmap for reform. By emphasizing trauma-informed organizational change, workforce equity, and systemic policy reform, the model supports the long-term retention of mental health professionals and ensures that care remains culturally responsive. Its relevance extends beyond mental health to social work and health care sectors, offering guidance on addressing workforce resilience and equity in broader contexts. Ultimately, the IWTR Model is a crucial tool for driving legislative action, educational redesign, and leadership development and fostering a more resilient and diverse workforce capable of addressing the growing global mental health crisis. To fulfill its potential, the model calls for an immediate and coordinated effort by policymakers, educators, and health systems to integrate trauma-informed care principles, equitably support providers, and develop leadership that reflects the diversity and resilience required to meet future challenges in mental health care [1,12,27].

## Figures and Tables

**Table 1:** Integrated Workforce Trauma and Resilience (IWTR) Model’s dimensions.

IWTR Dimension	Guiding Theories	Key Concepts
1. Impact of Trauma on the Mental Health Workforce	Trauma-informed care (TIC) Conservation of Resources (COR) Theory Intersectionality Theory	-Vicarious trauma, burnout, and compassion fatigue from repeated exposure to trauma. -Resource depletion (emotional, cognitive, organizational). -Additional burdens for BIPOC and LGBTQ+ providers (racial bias, discrimination, cultural taxation). -Moral injury due to systemic barriers.
2. Structural and Organizational Factors Affecting Workforce Retention	Job Demands–Resources (JD-R) Model Organizational Justice Theory	-Excessive job demands (caseloads, administrative burden, emotional labor). -Insufficient resources (pay equity, leadership support, wellness infrastructure). -Workplace fairness (equitable pay, workload distribution, transparent leadership). -Disparities in leadership representation and career advancement for marginalized providers.
3. Building Workforce Resilience Through Policy and Education	Trauma-informed care (TIC) Organizational Justice Theory Conservation of Resources (COR) Theory	-Trauma-informed education and training (resilience building, peer support, debriefing). -Trauma-informed workplace policies (leadership training, structured support services). -Legislative and systemic reforms (loan forgiveness, retention bonuses, reimbursement increases, safe staffing mandates).

**Table 2:** Policy and practice recommendations based on IWTR Model dimensions.

IWTR Model Dimension	Key Challenges Identified	Recommended Policy and Practice Interventions
1. Impact of Trauma on the Workforce	-Secondary trauma, burnout, compassion fatigue -Moral injury and resource depletion -Racial and intersectional inequities (BIPOC, LGBTQ+)	-Establish peer debriefing and supervision structures -Implement organizational trauma-informed policies -Equity-centered workforce initiatives (e.g., leadership pipelines for marginalized clinicians)
2. Structural and Organizational Factors	-High caseloads, low pay, limited leadership access -Administrative burden and lack of resources -Organizational injustice and exclusion from decision making	-Safe staffing mandates -Equitable pay policies and transparent promotion structures -Reduce administrative burdens (e.g., simplified documentation)
3. Building Workforce Resilience Through Policy and Education	-Lack of trauma-informed training and workforce preparation -Insufficient mental health investment -Workforce inequities impacting retention	-Embed trauma-informed care and resilience modules in training -Provide loan forgiveness and retention incentives -Increase funding for workforce development and leadership diversity

**Table 3:** Barriers to IWTR implementation and suggested solutions.

Potential Barrier	Description	Suggested Mitigation Strategies
Funding Limitations	Insufficient funding to implement new workforce policies or education reforms	-Advocate for state/federal mental health workforce investments -Leverage public–private partnerships -Use cost-savings data from reduced turnover as leverage
Leadership Buy-In	Resistance from leadership to adopting trauma-informed and equity-based changes	-Offer leadership training on trauma-informed and justice-oriented practices -Share data on workforce retention and quality outcomes to demonstrate value
Workplace Culture	Organizational resistance to systemic change; entrenched inequities	-Foster internal champions for change (e.g., diversity officers, mental health directors) -Gradually phase in trauma-informed interventions through pilot programs
Policy and Regulatory Barriers	Inflexible policies that inhibit innovation or equity reforms	-Engage policymakers early to align workforce needs with legislative priorities -Advocate for regulatory flexibility in workforce design
Lack of Diverse Leadership	Underrepresentation of marginalized groups in leadership	-Establish leadership pathways and mentorship programs for BIPOC, LGBTQ+, and immigrant clinicians -Set organizational diversity benchmarks tied to funding/incentives

## Data Availability

The literature used is in the reference list and is publicly available.
